# Aesthetic Management of Molar-Incisor Hypomineralization With Deep Resin Infiltration: A Case Report

**DOI:** 10.7759/cureus.85800

**Published:** 2025-06-11

**Authors:** Merieme Lferde, Hind Ramdi

**Affiliations:** 1 Department of Pediatric Dentistry, Faculty of Dental Medicine, Mohammed V University, Rabat, MAR

**Keywords:** case report, deep resin infiltration, esthetic management, icon, molar-incisor hypomineralization, white spot lesions

## Abstract

Molar-incisor hypomineralization (MIH) is a qualitative enamel defect that affects the first permanent molars and often the permanent incisors. In the anterior sector, MIH manifests as localized opacities in the coronal half of the tooth, typically well-defined, asymmetric, and ranging from whitish to brownish discolorations. These opacities cause significant aesthetic concerns for patients, affecting their self-esteem and social interactions. Effective management of these lesions can lead to a marked improvement in the patient's well-being.

However, the management of MIH presents significant challenges for clinicians. Indeed, these opacities are among the most problematic to treat among white spot lesions, with dental bleaching, resin infiltration, and composite restorations often proving insufficient to mask the lesion. In this context, the deep resin infiltration technique presents a promising solution. This method, in line with the therapeutic gradient, targets deeply localized lesions, such as those seen in MIH. It involves a slight micro-preparation of the enamel so that infiltration occurs exactly at the “ceiling” of the lesion. Through a clinical case report of a nine-year-old patient with MIH-related white opacities, we will describe the deep resin infiltration technique, illustrating its protocol and discussing its therapeutic considerations.

## Introduction

The condition known as molar-incisor hypomineralization (MIH) was initially described in 2001 by Weerheijm and colleagues, who described it as a "hypomineralization of systemic origin, presenting as demarcated, qualitative defects of enamel of one to four first permanent molars (FPMs) frequently associated with affected incisors" [1]. Recent findings have demonstrated that similar defects can also be present in other permanent teeth, as well as in primary teeth [2]. Clinically, MIH presents as demarcated opacities that vary in color from white to yellow-brown and have well-defined borders. These defects are the result of disruptions during the maturation phase of amelogenesis and may be associated with post-eruptive breakdown, hypersensitivity, and a significant aesthetic impact. The exact etiology of MIH remains unclear and is considered multifactorial. It results from systemic disturbances occurring during the maturation phase of amelogenesis, typically around the time of birth and the first four years of life. Proposed contributing factors include prenatal, perinatal, and early childhood illnesses such as respiratory infections, high fever, otitis media, gastrointestinal disturbances, and antibiotic use. Environmental influences, such as exposure to dioxins or bisphenol A, and nutritional deficiencies may also interfere with enamel mineralization.

MIH is a frequently encountered dental condition worldwide [3]. It holds significant clinical importance due to its high prevalence and its substantial consequences for the quality of life of affected children, impacting both functional and aesthetic aspects. The condition represents a considerable management challenge for practitioners, who must address the difficulties associated with accurate diagnosis and effective treatment.

Aesthetic concerns are particularly common in patients with MIH, especially when incisors are involved [4]. In these cases, an ultra-conservative approach is often preferred, as the volume and shape of the affected teeth are usually preserved. Traditional treatment modalities such as bleaching, microabrasion, and composite restorations have shown limited success in these cases. Bleaching often yields inconsistent results due to the altered optical properties of hypomineralized enamel. Microabrasion is effective only on superficial lesions and cannot address the deeper porosities characteristic of MIH. Composite restorations, while effective at masking discoloration, require mechanical preparation and the removal of healthy enamel, contrary to the principles of minimally invasive dentistry. To achieve this goal, the erosion-infiltration protocol was proposed. It involves superficial demineralization of the enamel to allow infiltration with a very fluid composite resin. However, the results were inconsistent, especially in MIH cases where the lesions are deeply embedded and covered by intact enamel. To address this limitation, the technique has been improved through the development of a new deep infiltration protocol proposed by Attal et al. [5].

This technique involves micro-invasive preparation of the affected tooth surface to allow optimal penetration of the resin infiltrant across the entire lesion. The aim of our article is to illustrate the step-by-step protocol of this technique in treating MIH-related white opacities, through a clinical case of a nine-year-old patient, and to highlight the key factors for the success of this therapeutic approach.

## Case presentation

**Figure 1:**
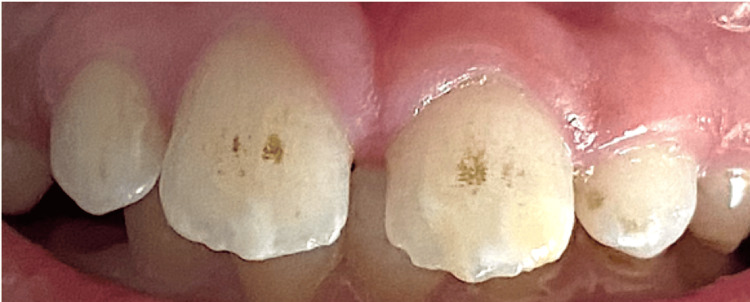
Intraoral view showing creamy white opacities with distinct borders and an asymmetric distribution between the homologous teeth on the buccal surface, associated with extrinsic black discolorations.

**Figure 2:**
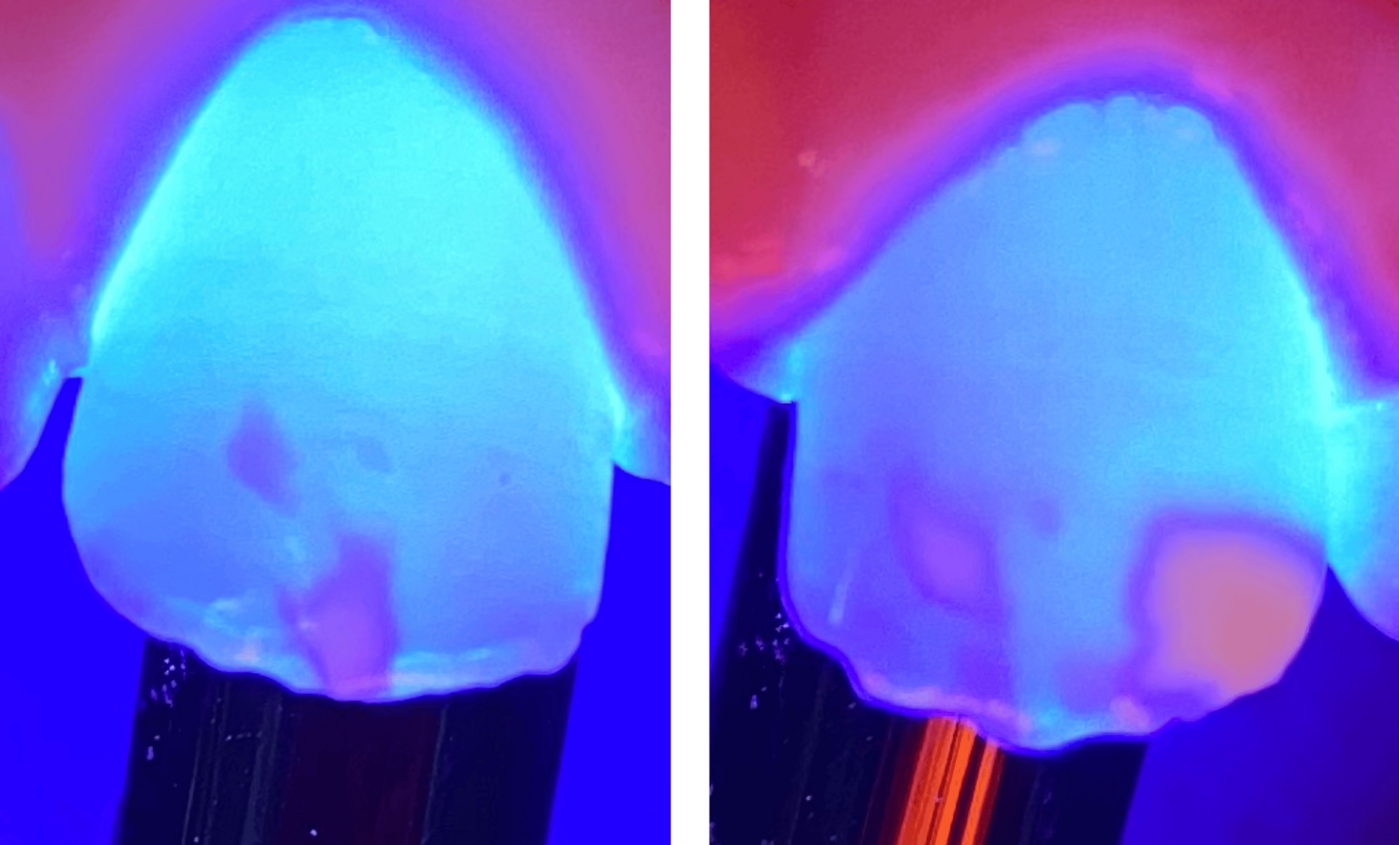
Lesion mapping by transillumination. The edges of the lesion, which appear more blurred, are more deeply embedded and would require more preparation to avoid a border effect. The opacity differential allows us to conclude that the lesion on the distal surface of tooth 21 is thicker than all the other lesions, making it more accessible for treatment.

A nine-year-old asthmatic patient, accompanied by his mother, presented to the Pediatric Dentistry Department at Ibn Sina Center for Consultation and Dental Treatment in Rabat, Morocco, with complaints of aesthetic concerns. He was not taking any medications at the time of consultation. Additionally, no familial history of enamel defects was reported by the parents. Clinical intraoral evaluation revealed well-defined white opacities with clear demarcation from the adjacent enamel on the buccal surfaces of the maxillary central incisors bilaterally, as well as on the left lateral incisor. These opacities were associated with superficial exogenous discoloration (Figure 1). Further clinical examination of upper permanent first molars showed atypical hypomineralization, supporting the diagnosis of MIH (Figure 2). The patient’s periodontal condition was normal, with no visible dental plaque.

A decision was made, under the agreement of the patient and his mother, to apply the deep resin infiltration technique on teeth 21 and 11, a minimally invasive approach for managing enamel white spots.

**Figure 3:**
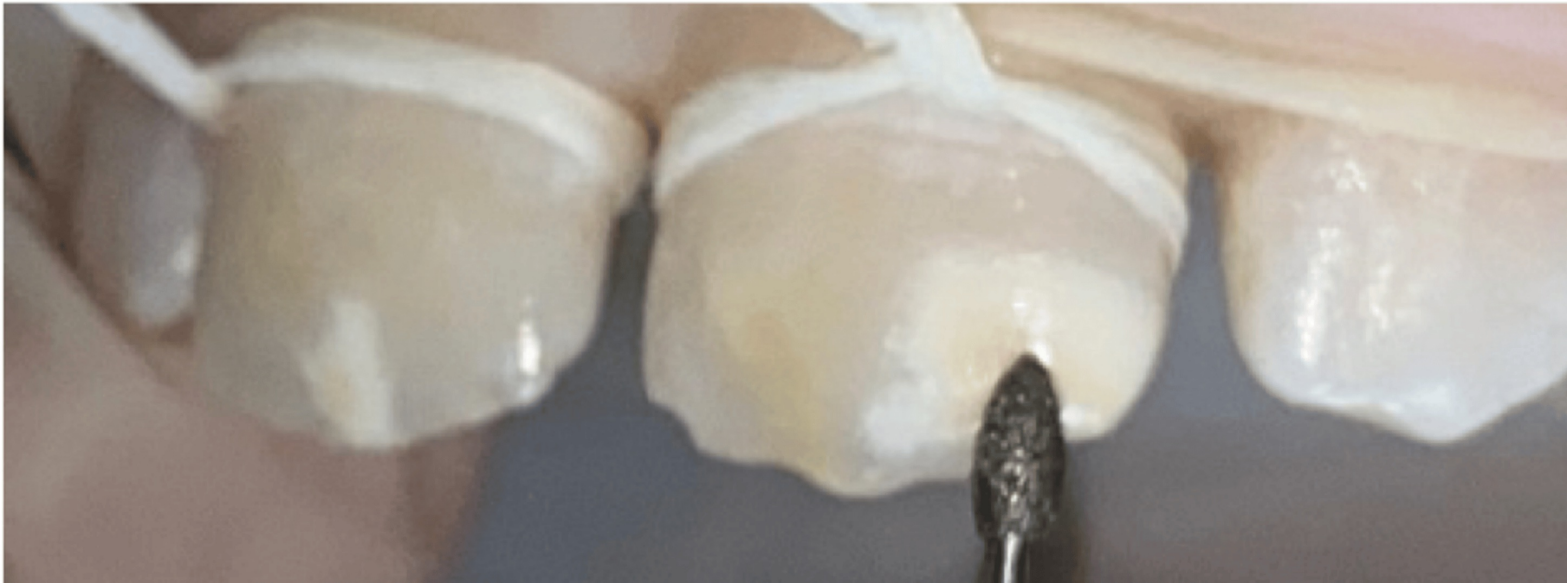
Exposure of the ceiling of the lesion (tooth 21) without completely removing it using a yellow-banded diamond bur at low speed under irrigation.

**Figure 4:**
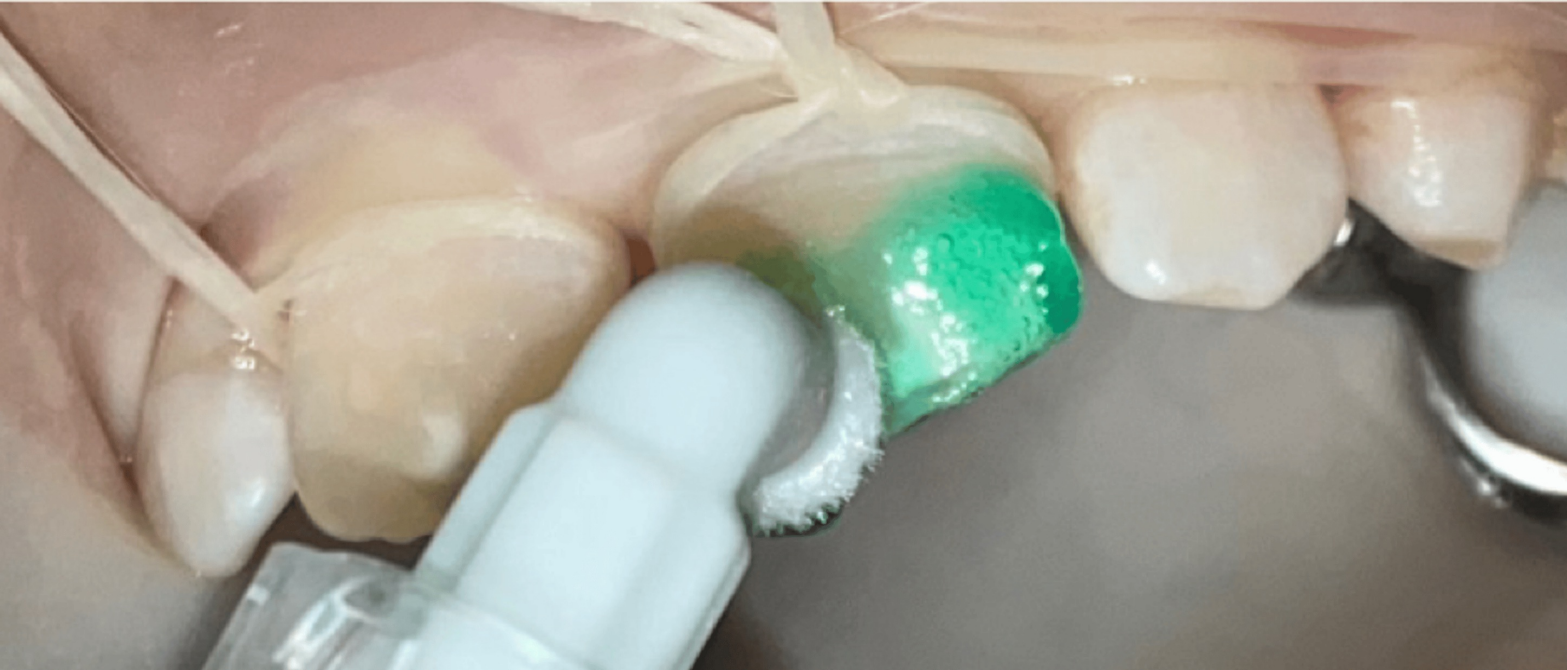
Opening the porosities of the enamel in the exposed areas after micro-etching by applying 15% hydrochloric acid (Icon-Etch ® DMG) and activating it for two minutes with a brush.

Teeth were first cleaned with a prophylactic paste during the initial session to eliminate the extrinsic discolorations. Afterward, a characterization of the spatial topography of the lesion was performed using transillumination (Figure 2). Next, under rubber dam isolation, minimal and selective milling with a yellow-band diamond bur at low speed allowed the exposure of the lesion ceiling (Figure 3). Following this, erosion was performed by applying a 15% hydrochloric acid gel (Icon-Etch, DMG, Hamburg, Germany) and activating it for two minutes (Figure 4). The acid gel was then carefully removed using a surgical suction device, followed by a 30-second rinse with a water spray. The enamel surfaces were then air-dried completely.

**Figure 5:**
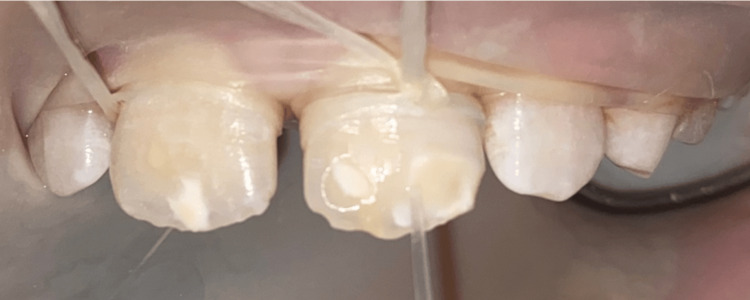
Validation of access to the ceiling (distal lesion of tooth 21): alcoholic draft with 99% ethanol (Icon-dry® DMG) for 30 seconds.

Prior to the infiltration of the lesion, an alcoholic draft with 99% ethanol (Icon-Dry® DMG) ensures that access to the lesion's ceiling is possible for resin infiltration into the body of the lesion. As the optical changes in the lesion were not satisfactory, an additional cycle was decided (selective additional micro-milling at the periphery, erosion, alcoholic draft) (Figure 5). A second alcoholic draft confirmed that the lesion was ready for infiltration.

**Figure 6:**
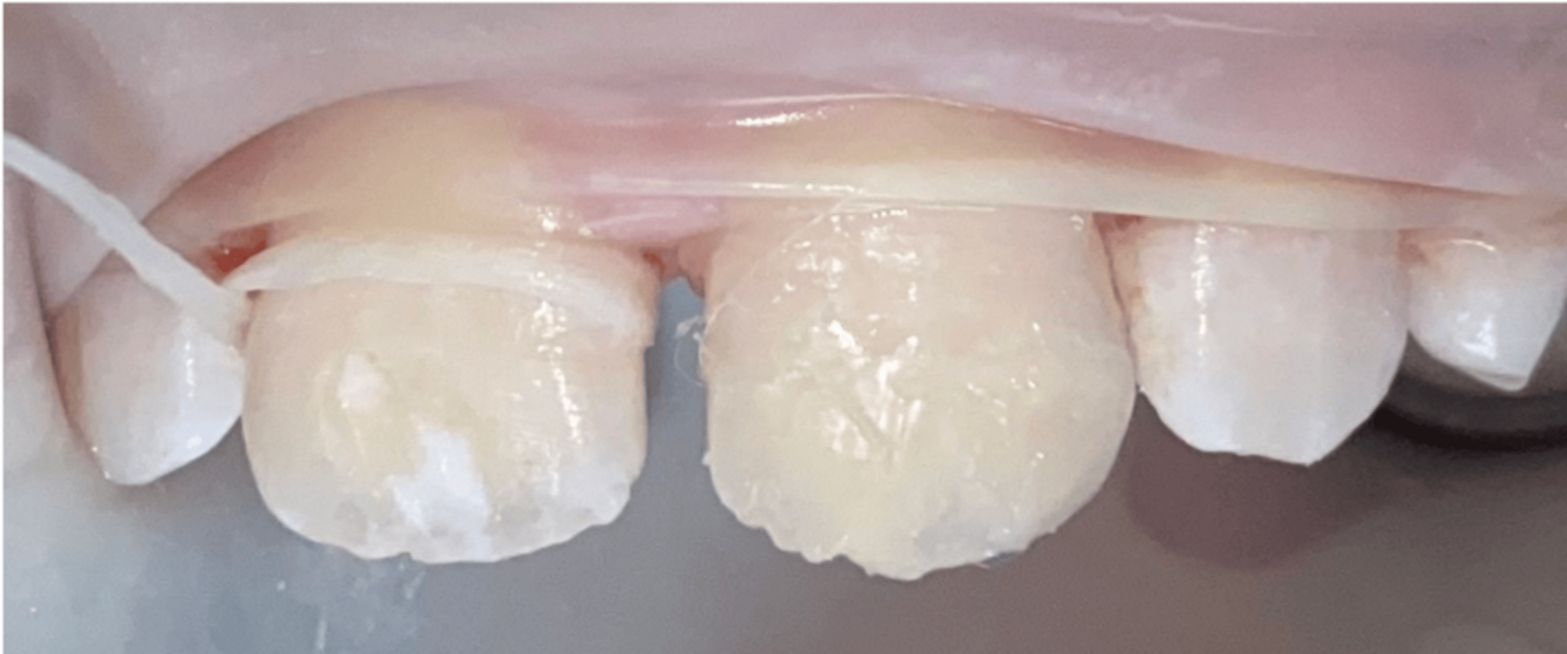
Application of the fluid resin (Icon Infiltrant® DMG).

**Figure 7:**
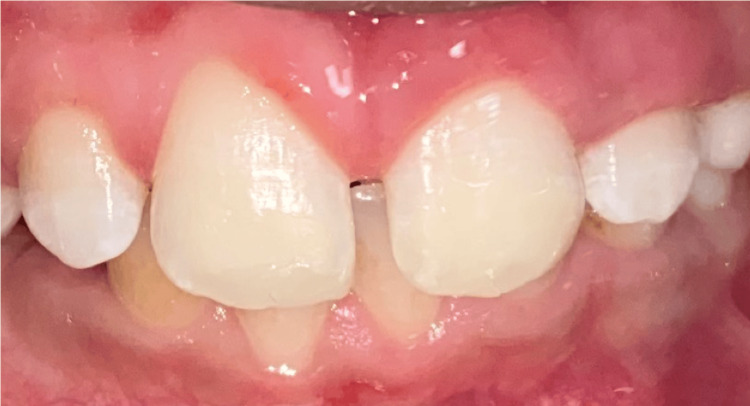
The same steps are repeated for tooth 21. Final result after polishing the surface.

The resin infiltrant (Icon Infiltrant® DMG) was then applied. The product was vigorously rubbed with an applicator for three minutes, followed by light curing for 40 seconds. A second layer of infiltrant was applied, followed by another 40-second light curing. Next, the composite was placed directly after the second infiltration to restore the lost substance (Figure 6). The same steps were repeated for tooth 11. After removing the rubber dam, the tooth surfaces were polished with flexible abrasive discs and silicone polishers to achieve a smooth and natural appearance. A significant aesthetic enhancement was achieved immediately after the procedure, with complete coverage of the discoloration (Figure 7).

## Discussion

MIH is an enamel development anomaly that is defined as a qualitative defect of systemic origin, affecting at least one of the first permanent molars (FPM) and, in some cases, the permanent incisors (PI) [1]. It is a prevalent condition affecting up to 40% of the population and is therefore a major public health problem with significant repercussions on dental health, functional well-being, and aesthetic outcome [3].

Regarding the diagnosis of MIH, a set of criteria has been established by Weerheijm et al. [6], two of which were observed in our patient. First, the patient exhibited well-defined enamel opacities with a clear contrast between the affected and healthy enamel. The opacities were asymmetrical in the homologous teeth. Regarding their color, they can vary from creamy-white to yellow-brown [6]. In our patient, they were creamy-white, suggesting a less severe enamel alteration, as darker shades typically indicate a more significant change in enamel composition. The opacities were typically located in the upper two-thirds of the incisal surface, making them visible when the patient smiled and raising significant aesthetic concerns. Second, we observed post-eruptive enamel breakdown (PEB) on teeth 26 and 36. This occurs as porous enamel is more susceptible to chipping, especially under the influence of masticatory forces, further aligning with the diagnostic criteria for MIH.

These PEBs are less common anteriorly, as anterior teeth are less severely disrupted and are not typically subjected to significant occlusal forces on the labial surface. However, an unattractive dental appearance frequently motivates patients to pursue aesthetic treatments due to the impact on their social interactions and self-esteem [7].

Therefore, whenever the volume and shape of the affected teeth are preserved, as in our case, invasive methods are avoided. In this context of tissue preservation, the practitioner must choose a treatment adapted to the clinical situation, following a therapeutic gradient from the most conservative to the least conservative [8]. It is within this framework that treatments such as the erosion-infiltration technique were initially proposed. However, the aesthetic results of this technique have been unpredictable and rarely successful [5]. This highlights the need for another important consideration for adequate and predictable aesthetic treatment in MIH cases: adopting a three-dimensional approach to the lesion. Indeed, the anatomopathology of the lesion in MIH is challenging: it begins at the amelo-dentinal junction rather than at the surface. Since these lesions are deeply embedded and covered by intact enamel, treatments like erosion-infiltration may fail to produce a favorable optical effect. To overcome this situation, Attal et al. suggested the concept of deep infiltration, which involves a slight alteration of the enamel through micro-preparation, ensuring that the infiltration reaches the "ceiling" of the lesion [5].

The protocol followed in our patient is based on three steps: (1) accessing the ceiling of the lesion, (2) infiltrating the lesion, and 3) restoring the substance loss.

Accessing the ceiling of the lesion

To effectively guide access to the lesion’s ceiling, a clear understanding of its spatial topography is essential. Transillumination is a simple, non-invasive, and painless method that proves useful for mapping white enamel lesions in anterior teeth. For this, a mapping is established by directing UV light from a photopolymerizing lamp perpendicularly to the palatal surfaces [9]. The light passes through the dental structure until it reaches the outer tissue, allowing for the analysis of two key image parameters: opacity and sharpness [5]. The greater the opacity of the lesion, the thicker it is, requiring less preparation for access, as was the case for the distal lesion of tooth 21. Conversely, the more blurred the lesion appears, the deeper it lies, as was the case for the edges of all the spots, which required more extensive preparation to reach them. The preparation can be done either with micro-abrasion using aluminum oxide (50 microns) or milling with a yellow-ring burr at low speed, as performed on our patient.

After the guided micro-preparation, surface erosion is performed using hydrochloric acid gel (15%). Hydrochloric acid is preferred over phosphoric acid as the penetration depth of hydrochloric acid etching is more than twice that of phosphoric acid gel (37%), allowing for deeper penetration into the lesion [10].

The main challenge of this therapeutic approach lies in determining the optimal moment to infiltrate the enamel opacity. This is a key step that determines the success of the outcome. This condition is assessed using the alcohol test (ethanol) (Icon-Dry, DMG), which, due to its high refractive index, helps predict the outcome after resin infiltration. After applying the alcohol for 30 seconds, areas that transition from opaque to translucent can be considered successfully accessible [5]. However, if no change is observed or only in part of the lesion, it indicates that the lesion has not been sufficiently reached in depth, necessitating selective re-treatment focused on the remaining opaque areas. In our case, an additional selective cycle on the areas that did not undergo optical modification was required to achieve a satisfactory result.

Infiltrating the lesion

Resin infiltration is performed only after confirming the success of the previous step [11]. The infiltrating resin is primarily composed of triethylene glycol dimethacrylate (TEGDMA), which makes up about 78% of the formula. This low-viscosity, highly fluid resin monomer has a refractive index (1.52) similar to that of sound enamel (1.62). It enhances the light transmission through hypomineralized enamel, thereby restoring its transparent appearance [5]. TEGDMA is the monomer of choice for this method, as it has been shown to penetrate more efficiently compared to bis-GMA (bisphenol A-glycidyl methacrylate) [12].

In addition to the optical improvements, resin infiltration provides physical benefits to the hypomineralized enamel. Paris et al. [13] observed improved resistance to demineralization and microhardness. It is due to the resin's ability to penetrate the interprismatic enamel and form a reinforced resin-enamel tissue, known as the hybrid layer, which is distinct from dentin hybrid layers due to the absence of collagen fibers [14].

The infiltration is done initially for two minutes, avoiding the light from the operating lamp and the diodes of the binocular loupes to ensure maximum diffusion of the resin. Since the resin is primarily a hydrophobic monomer, it is essential to eliminate as much residual water as possible within the lesion through the alcoholic step [12].

The underlying concept of resin infiltration is to fill the hypomineralized enamel with resin through capillary forces. The resin is applied for three minutes, and the longer it is applied, the deeper the diffusion will be [15]. After gently air-blowing to remove excess resin, a 40-second photopolymerization is performed. Then, the resin is applied a second time for 15 seconds. The time for this second infiltration is reduced because its sole purpose is to compensate for the polymerization shrinkage, rather than to diffuse deeper.

While resin infiltration offers optical enhancements by increasing diffusion rates and improving surface hardness, it has been shown to be more prone to discoloration when compared to other alternatives. Consequently, patients must practice increased vigilance regarding home hygiene and dietary awareness. It is important to note that this issue primarily arises in instances of superficial infiltration, as in deep infiltration, the resin infiltrant remains isolated from the oral environment [5].

Restoring the substance loss

A thin layer of enamel-mass composite is placed to fill the small concavities resulting from the chemical and mechanical treatments of the lesions. The infiltrating resin acts as an adhesive, so the composite is placed directly after the second infiltration. Several studies have shown that infiltration resins exhibit strong adhesive performance when bonded to composite materials [16].

Research evaluating the durability of aesthetic outcomes indicates consistent benefits for at least six months [17]. Although resin infiltration is a conservative aesthetic procedure that respects tooth structure, it does come with certain drawbacks. Indeed, biofilm and organic residues adhering to the surfaces may block the pores within the lesions, preventing complete resin penetration and ultimately limiting the effectiveness of the procedure. Additionally, resin infiltration is not effective against pigmented spots. In these cases, vital nightguard bleaching is an effective method for converting a pigmented area into a white one. After whitening, a two-week waiting period is recommended before conducting resin infiltration to prevent interference with the curing process [18].

In cases where whitening is not feasible (such as in children under 18 years or due to financial constraints), an alternative method involves rinsing with Icon-Etch, followed by the application of 5% sodium hypochlorite for five minutes (with renewal of the solution). This process helps eliminate some of the proteins responsible for discoloration, although it is much less effective than whitening and may require several applications of Icon-Etch.

Given its high prevalence and potential for significant impact, an appropriate therapeutic approach to MIH holds substantial clinical importance. The case presented demonstrated a better balance between achieving aesthetic results and maximizing tissue preservation. Each step in the process, from diagnosing the lesion to applying resin infiltration, is grounded in fundamental knowledge of the lesion's anatomopathology, the mechanisms of diffusion and lightening, and the mechanical and optical properties of the materials used.

## Conclusions

Erosion-infiltration is an aesthetic and conservative approach that effectively manages white spot lesions on enamel by masking rather than eliminating the stain. This technique leads to a noticeable improvement in aesthetic outcomes and enhances the patient's satisfaction. To achieve these outcomes, a rigorous therapeutic strategy is essential. The multi-step process includes mechanical abrasion for effective access to the lesion through micro-milling, chemical erosion using 15% hydrochloric acid to open the enamel porosities, infiltration of a very fluid resin into these porosities, and subsequent repair of the thin layer of lost enamel using a composite material.
